# On the Effect of Soft Molecularly Imprinted Nanoparticles Receptors Combined to Nanoplasmonic Probes for Biomedical Applications

**DOI:** 10.3389/fbioe.2021.801489

**Published:** 2021-12-21

**Authors:** Nunzio Cennamo, Alessandra Maria Bossi, Francesco Arcadio, Devid Maniglio, Luigi Zeni

**Affiliations:** ^1^ Department of Engineering, University of Campania Luigi Vanvitelli, Aversa, Italy; ^2^ Department of Biotechnology, University of Verona, Verona, Italy; ^3^ BIOtech Center for Biomedical Technologies, Department for Industrial Engineering, University of Trento, Trento, Italy

**Keywords:** nanoMIPs, nanoplasmonics, optical chemical sensor, molecularly imprinted polymers, nanograting

## Abstract

Soft, deformable, molecularly imprinted nanoparticles (nanoMIPs) were combined to nano-plasmonic sensor chips realized on poly (methyl methacrylate) (PMMA) substrates to develop highly sensitive bio/chemical sensors. NanoMIPs (d_mean_ < 50 nm), which are tailor-made nanoreceptors prepared by a template assisted synthesis, were made selective to bind Bovine Serum Albumin (BSA), and were herein used to functionalize gold optical nanostructures placed on a PMMA substrate, this latter acting as a slab waveguide. We compared nanoMIP-functionalized non-optimized gold nanogratings based on periodic nano-stripes to optimized nanogratings with a deposited ultra-thin MIP layer (<100 nm). The sensors performances were tested by the detection of BSA using the same setup, in which both chips were considered as slab waveguides, with the periodic nano-stripes allocated in a longitudinal orientation with respect to the direction of the input light. Result demonstrated the nanoMIP-non optimized nanogratings showed superior performance with respect to the ultra-thin MIP-optimized nanogratings. The peculiar deformable character of the nano-MIPs enabled to significantly enhance the limit of detection (LOD) of the plasmonic bio/sensor, allowing the detection of the low femtomolar concentration of analyte (LOD ∼ 3 fM), thus outpassing of four orders of magnitude the sensitivies achieved so far on optimized nano-patterned plasmonic platforms functionalized with ultra-thin MIP layers. Thus, deformable nanoMIPs onto non-optimized plasmonic probes permit to attain ultralow detections, down to the quasi-single molecule. As a general consideration, the combination of more plasmonic transducers to different kinds of MIP receptors is discussed as a mean to attain the detection range for the selected application field.

## 1 Introduction

Optical sensors hold great potential in the bioanalytical field, finding increasing point-of-care (POC), and Internet-of-things (IoT) applications. Optical sensing enables to achieve rapid and automated measurements in many areas, from bioanalysis to clinical medicine, from industrial applications to security, and from environmental monitoring to food applications (Loncar et al., 2000; Horváth et al., 2003; Mukundan et al., 2009; Hulme et al., 2011; Schmitt et al., 2011; Wang et al., 2012; Gao et al., 2018; Barshilia et al., 2020; Fuentes et al., 2020). In particular, the optical transducers based on Surface Plasmon Resonance (SPR) and Localized Surface Plasmon Resonance (LSPR) phenomena enable to achieve excellent performances in the above applications (Harris and Wilkinson 1995; Huang et al., 2006; Shinbo et al., 2011; Baryshev et al., 2013; Cennamo et al., 2017; Zheng et al., 2017; Chen et al., 2018; Cennamo et al., 2019a).

Several are the optical bio/sensor configurations that have been realized through optical fibers or planar waveguides. Optical sensors integrated on silicon-based substrates find uses in gas sensing (Siebert and Müller, 2005; Airoudj et al., 2008; Huang et al., 2014; Consani et al., 2018; Maurya et al., 2018), magnetic field sensing (Deghdak et al., 2017; Ge et al., 2021), detection of biological, and chemical species (Densmore et al., 2007; Mukundan et al., 2009; Schmitt et al., 2011; Kozma et al., 2014; Kumar et al., 2021). Moreover, these sensors offer all the advantages of the technological and fabrication processes typical of the microelectronic industry, such as the integration with other components, the low-cost, and the small-size of the produced device. The main disadvantages of the silicon-based approach are related to issues concerning the realization of thick layers, despite several efficient solutions have been proposed over the last years (White 2017). In particular, the use of multimodal optical waveguides (achievable using thick layers) instead of monomodal ones improves the performances of plasmonic sensors. The number of modes depends on the waveguides layer thickness and plays a fundamental role in the sensor’s performance, hence, over the last years, multimodal waveguides have been gaining preference, as these offer a better sensitivity than monomodal waveguides (Slavik et al., 2001; Dwivedi et al., 2008; Kanso et al., 2008; Suzuki et al., 2008; Gupta and Verma 2009; Gasior et al., 2014), despite their non-optimal signal-to-noise ratio (SNR). The trade-off between SNR and sensitivity has mainly been investigated by using large core diameter optical fibers instead of monomodal ones (Slavik et al., 2001; Dwivedi et al., 2008; Suzuki et al., 2008; Gupta and Verma 2009). Similarly, multimode slab waveguides have also drawn the scientific community’s attention so far, showing excellent performances and results (Wang et al., 2012; Mishra et al., 2016; Cennamo et al., 2017; Walter et al., 2020). Earlier, we proposed a multimode slab waveguide based on a poly (methyl methacrylate) (PMMA) substrate and intended for the realization of plasmonic biosensors with a very simple experimental setup. This PMMA chip has been showing to perform as an SPR sensor, as reported in (Cennamo et al., 2017). Moreover, such SPR probe has also been successfull for different biochemical sensing applications when exploiting specific biomimetic receptors called molecularly imprinted polymers (MIPs) (Zeni et al., 2018; Arcadio et al., 2021a). MIPs are tailor-made polymeric receptors prepared by a template assisted synthesis (Arshady and Mosbach 1981). The use of MIPs in optical sensing offers a number of advantages, such as the possibility to prepare the MIP receptor customized for the capture of any kind of target analyte, from small analytes to proteins (Bossi et al., 2007; Cennamo et al., 2018a), to cell and viruses (Eersels et al., 2016; Piletsky et al., 2020). Moreover, MIPs are cheap to synthesize and far more robust in withstanding solvents and temperature changes than biological receptors. Among the key advantages of MIPs is the possibility to prepare them in different formats. MIPs in the form of thick layer can be obtained starting from a pre-polymerized solution that is spin coated on the plasmonic sensor (Schmidt et al., 2004; Cennamo et al., 2016; Pesavento et al., 2021), this yields to layers of about 300–800 nm. Alternatively, MIP thin layers, in the range of one to few hundreds of nm, can be formed starting from monomers polymerized directly onto the optical transducer (Cennamo et al., 2018a; Özgür et al., 2020). By exploiting a bottom up synthesis, in which an initiator or an iniferter is coupled to the transducer’s surface, the polymerization starts just at and from the surface, thus yielding to ultrathin MIP layers (less than 100 nm) (Bossi et al., 2010; Gupta et al., 2011). At last, MIPs can be synthesized in the form of nanoparticles, called nanoMIPs and sometimes referred to as plastic antibodies, which are characterized by sizes from 10 to about 100 nm, high surface to volume ratio, fast mass transfer kinetics, and a limited number of binding sites per nanoparticle (Poma et al., 2010; Cenci et al., 2018a). NanoMIPs have been successfully used to functionalize the optical transducer, including PMMA plasmonic platforms (Cennamo et al., 2020). The state of art of MIP-based photonic structures has been recently discussed (Chiappini et al., 2020).

Whether the plasmonic sensors performances are affected by the different MIP formats is a question worth exploring. Moreover, simple sensor configurations with high sensitivity are key requisites for bio/chemical sensing applications, where the needed limits of detection (LODs) for the analyte are often below the picomolar.

To achieve very low LOD values, the optical bio/chemical sensors can be modified either at the level of optical sensor’s sensitivity, or at the receptor’s level, or at both.

Along these lines, earlier in our group, we demonstrated that an SPR probe based on a PMMA slab waveguide for the detection of furfural and using a specific MIP receptor layer, having a thickness of about 700 nm (Zeni et al., 2018) has an overall performance similar to that obtained for the transducer being an SPR D-shaped Plastic Optical Fiber (POF) sensor functionalized with the same MIP layer (Cennamo et al., 2011).

We expect information on the effects of the MIP formats can be devised by the comparison between nano-plasmonic probes based on optimized nanostructured slabs (Arcadio et al., 2021b; Arcadio et al., 2021c) and non-optimized plasmonic probes (Cenci et al., 2018b; Cennamo et al., 2020), ultimately enabling to modulate the sensor’s response.

To date, in order to improve the optical response of the plasmonic sensors based on PMMA multimode slab waveguides, we have been exploiting the maximum potential of such a chip and have been taking full advantages of its dimension (10 mm × 10 mm × 0.5 mm), fully compliant to the modern holder of electron beam lithography (EBL) systems (e.g., Zeiss Supra v35—Raith Elphy Quantum system), so to realize nanopatterns suitable for a nanoplasmonic sensor. In particular, we developed and tested nanoplasmonic sensors based on gold nanograting fabricated on the top of PMMA chips (Arcadio et al., 2021b; Arcadio et al., 2021c).

We realized and tested several nanogratings with different nanopatterns to show the sensor’s sensitivity variation *via* numerical and experimental results (Arcadio et al., 2021b). More specifically, to determine the role of the grating in the plasmonic phenomenon, periodic, and non-periodic configurations have been studied to show how an optimized sensor, in terms of bulk sensitivity, can be obtained exploiting a specific periodic nanopattern (Arcadio et al., 2021b). Additionally, the same PMMA nano-plasmonic sensor chips were monitored exploiting two different experimental configurations, both produced by our research group (Arcadio et al., 2021b; Arcadio et al., 2021c), where the PMMA substrate was considered as a transparent substrate in (Arcadio et al., 2021b), or a multimode waveguide in (Arcadio et al., 2021c), as in the present work. In the cases studied as a proof of concept, we functionalized the nano-grooved surface with a specific nanolayer of a MIP selective for bovine serum albumin (BSA) detection (Arcadio et al., 2021b; Arcadio et al., 2021c).

Here we report a PMMA nanoplasmonic sensor chip, with a bulk sensitivity lower than that reported for optimized nanoplasmonic sensors (Arcadio et al., 2021b), and combined to soft highly responsive nanoMIP receptors for the detection of BSA. We aimed at demonstrating how the bio/chemical sensors’ performance can be modulated or improved by a strategy based on using highly responsive receptors combined to non-optimized nanoplasmonic chips. It is anticipated that without stressing the capabilities of production technologies, in terms of high performances of the plasmonic probe, extremely high sensitive bio/chemical sensors could be attained, just by choosing the proper kind of receptor, such as soft nanoMIPs. Moreover, the combination of plasmonic platforms to different MIP receptors results in the ability to tune the sensor’s detection range over 10 orders of magnitude.

## 2 Materials and Methods

### 2.1 Nanoplasmonic Sensor Chip

**FIGURE 1:**
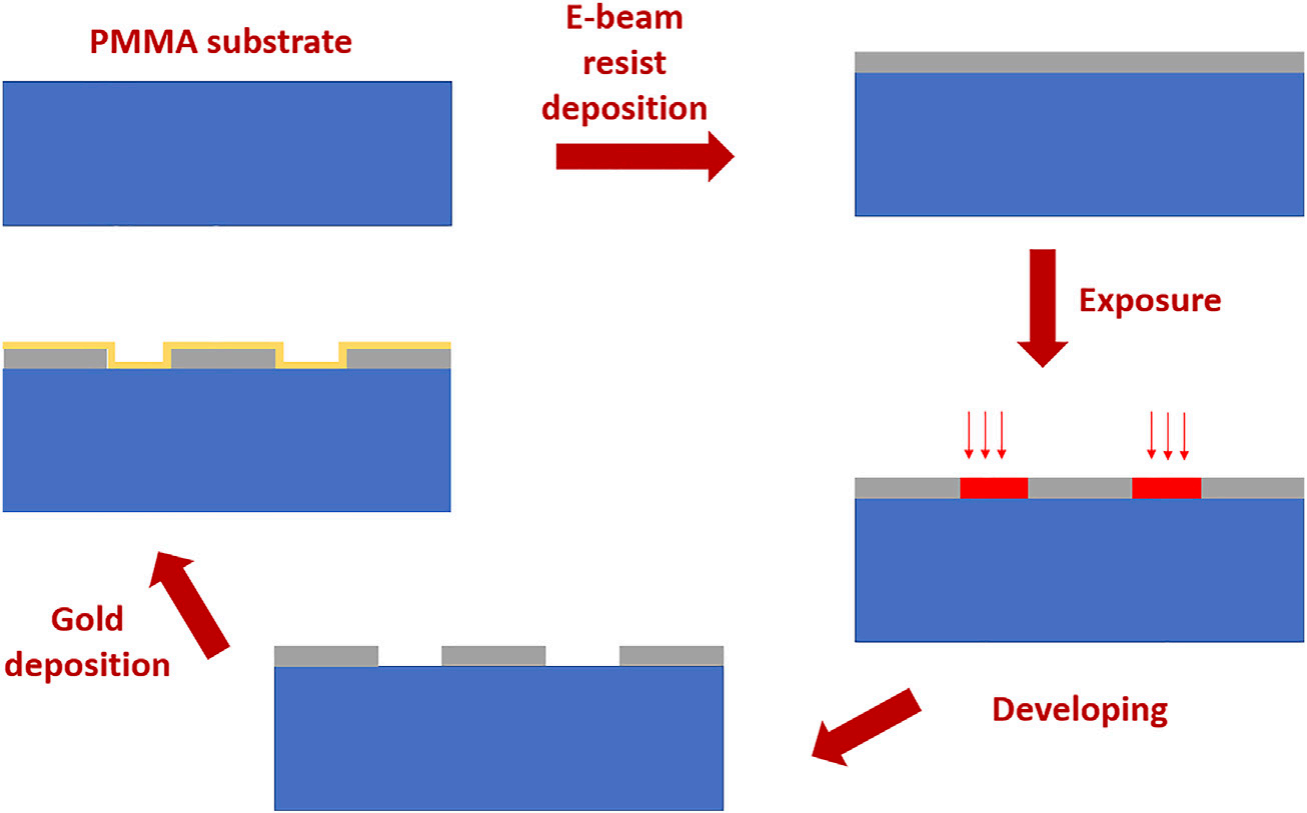
Fabrication steps to realize the gold nanograting based on EBL.

The plasmonic sensor chip is based on gold nanograting (GNG) realized on a PMMA substrate. The sensor’s fabrication is reported in Figure 1, showing step by step the production process based on an electron beam lithography (EBL). In particular, the initial chip consists of a 10 mm × 10 mm × 0.5 mm PMMA layer (GoodFellow, Huntingdon, England).

**FIGURE 2:**
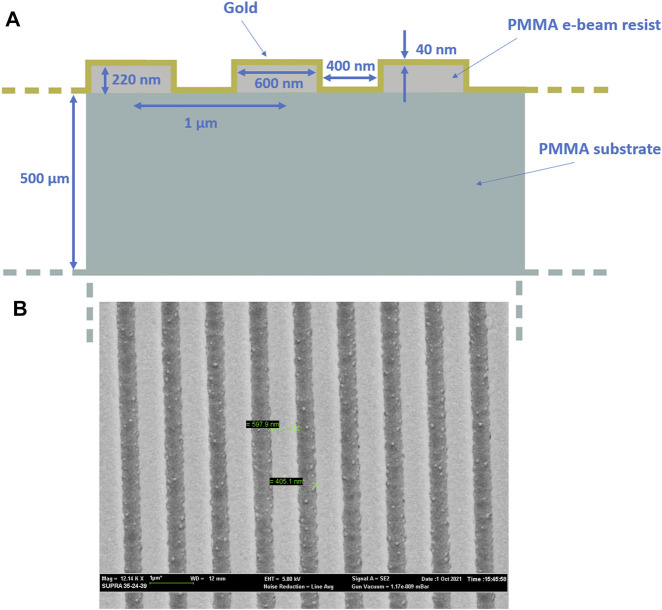
**(A)** Cross-section of the GNG. **(B)** SEM image of the GNG.

This PMMA chip is the substrate of the nanoplasmonic sensing surface. On the PMMA chip a 220 nm thick positive PMMA e-beam resist (AR-P 679.04, AllResist GmbH, Strausberg, Germany) layer has been realized by a spin coater machine. An EBL system (Zeiss Supra v35—Raith Elphy Quantum) has been used to obtain the nanograting pattern (by setting the EBL parameters to: acceleration voltage of 20 kV, a 7.5 μm aperture and a beam current of 20 pA). After the development process, on the realized nanopattern, a gold nano-film with a thickness of 40 nm is deposited by a sputter coater machine (BalTec SCD 500, Schalksmühle, Germany). We have already used this fabrication process for all the analyzed sensor configurations reported in (Arcadio et al., 2021b), changing the exposed pattern. In this work, we have realized the not-optimized nanopattern reported in Figure 2, where we have also reported an outline of the cross-section together with the SEM image of the fabricated gold nanograting. The dimensions of the GNG are reported in Figure 2A; the pattern showed a bulk sensitivity value of about a half that obtained by an optimized pattern and reported in (Arcadio et al., 2021b).

### 2.2 Experimental Setup

**FIGURE 3:**
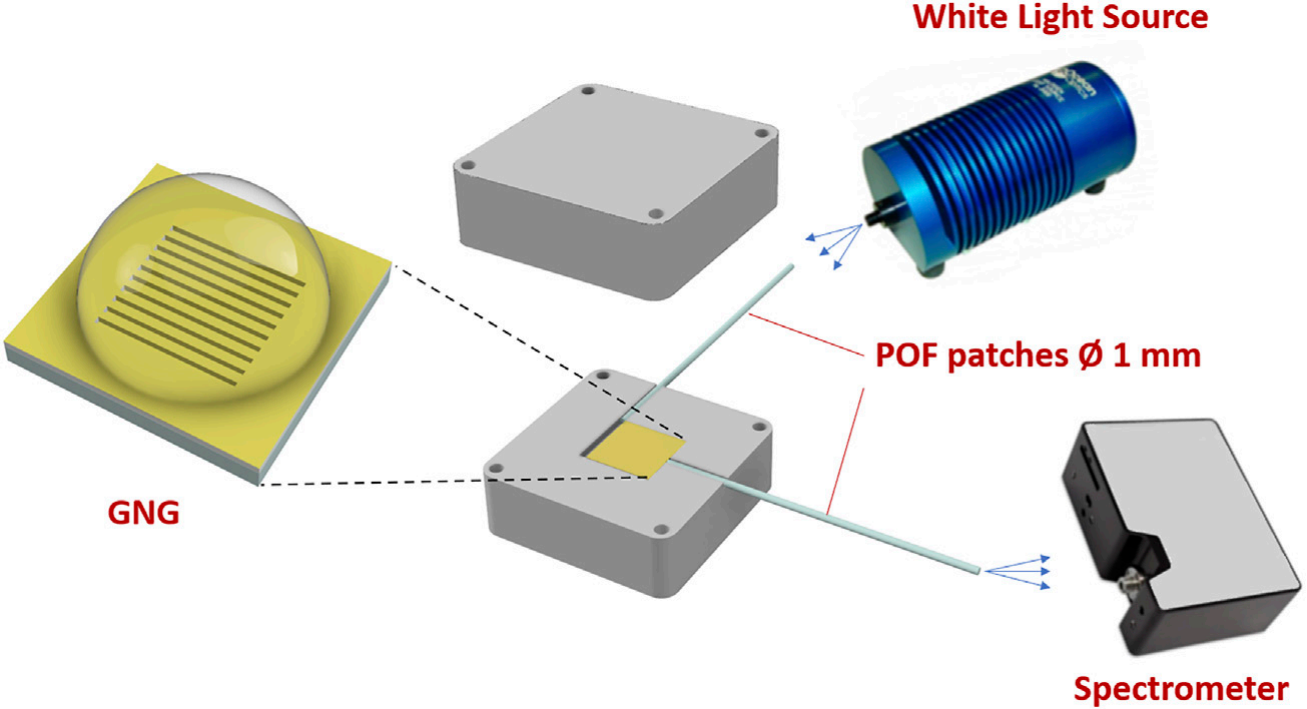
Nanoplasmonic bio/sensor chip’s experimental setup.

In this work, to monitor the nanoplasmonic sensor chip, we used an experimental setup where the PMMA substrate acts as a slab multimode waveguide to excite the nanoplasmonic phenomenon on the sensing nanostructured surface. The equipment consists of a white light source (HL-2000-LL, manufactured by Ocean Optics, Dunedin, FL, United States, with an emission range from 360 to 1700 nm), a spectrometer with a detection range from 350 to 1,023 nm (FLAME-S-VIS-NIR-ES, manufactured by Ocean Optics, Dunedin, FL, United States), two POF patches, and an aluminium holder realized by our group to monitor this kind of plasmonic chip (Cennamo et al., 2017). As shown in Figure 3, all these components are connected similarly to the PMMA plasmonic sensors reported in (Cennamo et al., 2017; Arcadio et al., 2021c). Figure 3 shows the light propagation path through the setup, with the light propagated from the source to the first plastic optical fiber (POF) patch (1 mm total diameter). At the end of this POF, a trench of air in the custom holder is used to enlarge the number of angles useful to excite plasmons in the nanostructured gold surface of the PMMA slab waveguide. On the other hand, another POF patch (1 mm total diameter) kept at the end of the PMMA-gold waveguide, at a 90° angle with respect to the air trench, collects the transmitted light through the sensor chip to direct it towards the spectrometer.

### 2.3 Chemicals and Materials

Acrylamide (Aam), Methacrylic acid (MAA), N-tert-butylacrylamide (TBAm), N,N′-methylenebisacrylamide (BIS), N,N,N′,N′-tetramethylethylenediamine (TEMED), ammonium persulfate (APS), sodium dodecyl sulfate (SDS), sodium dihydrogen phosphate, sodium monohydrogen phosphate, sodium chloride, hydrochloric acid, Trizma^®^ base, 2-[N-morpholino]ethanesulphonic acid (MES), N-(3-dimethylaminopropyl)-N′-ethyl-carbodiimide hydrochloride (EDC), N-hydroxysulfosuccinimide (NHS), sodium acetate, Tween-20, ethanol, bovine serum albumin (BSA), k-casein, horseradish peroxidase (HRP), and 3,3′,5,5′-tetramethylbenzidine (TMB) were from Sigma-Aldrich (Darmstadt, Germany).

### 2.4 Molecularly Imprinted Polymer Nanoparticles

Molecularly imprinted nanoparticles (nanoMIPs) were synthesized as reported in (Cennamo et al., 2020). Briefly, Acrylamide (Aam), methacrylic acid (MAA), N-tert-butylacrylamide (TBAm) were added at 8, 8, and 4% (mol/mol) respectively, together with 80% (mol/mol) of N, N′-methylenebisacrylamide (BIS) in 20 mM phosphate buffer (PB) pH 7.4 supplemented with SDS 0.02% (w/v). The template bovine serum albumin was added to the nanoMIP-vials to the final concentration of 1.2 µM. Vials were closed with rubber caps, sonicated for 10 min and bubbled with N_2_ for 15 min. Then APS (0.04% w/v) and TEMED (0.03% w/v) were added and the polymerization was carried out at 20°C for 20 h. Control, non-imprinted (nanoNIP) nanoparticles were synthetized using the same protocol but in the absence of the template. At the end of the polymerization the pH was adjusted to 8 with 50 mM Trizma-base, Trypsin was added to the solutions in a 1:25 (w/w) ratio with respect to the template, and incubated for 2 h at 37°C. The nanoMIPs were then dialyzed against 3 × 3 L of pure water. The yield of polymerization, calculated from the weight of the lyophilized nanoMIPs with respect to the total weight of the monomers added to the synthetic batch, was 90%. The nanoMIPs mean diameter was estimated by dynamic light scattering and resulted as d = 30 nm and had a polydispersity index (PDI) of 0.192, in accordance with (Cenci et al., 2018b); the profile of the nanoMIP size distribution is accessible in the Supplementary Information (Supplementary Figure S1).

### 2.5 Functionalization of the Nanoplasmonic Sensing Chip With NanoMIPs

**FIGURE 4:**
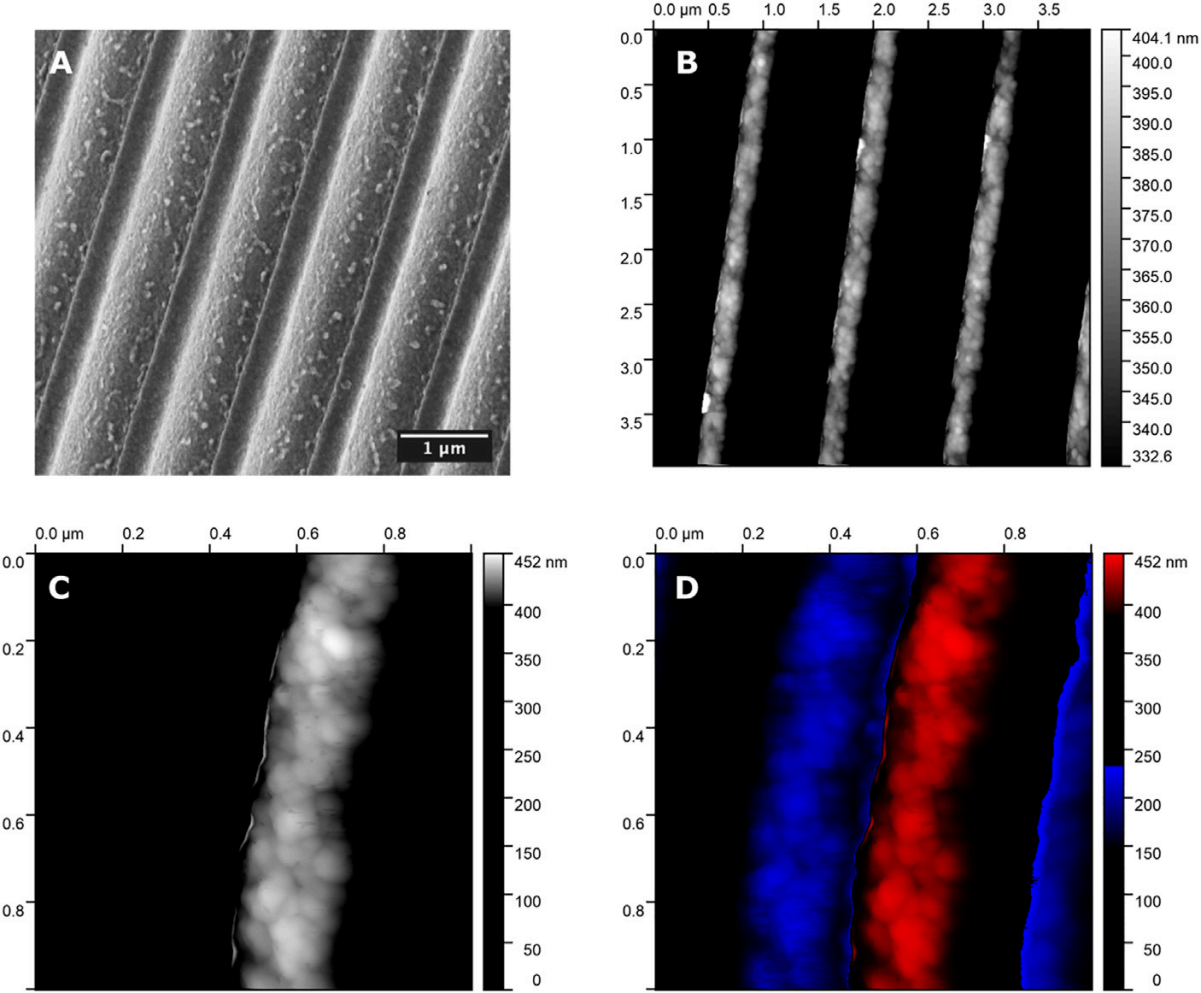
**(A)** SEM image of the nanoMIP’s functionalized nanograting. AFM images of the nanoMIP’s of the functionalized nanograting: **(B)** Top view of the nanoMIP-nanograting; **(C)** expanded view of the top of one single nanostripe with particular focus on the nanoMIPs (mean d = 30 nm) coupled to the nanostripe; **(D)** expanded view onthe top of one nanostripe, depicted in red, with evidence on the coupled nanoMIPs and flanked by the two valleys of the nanograting structure, that are represented in blue.

Plasmonic nanogratigs were functionalized with nanoMIPs, according to the protocol reported in (Cennamo et al., 2020). Briefly, after plasma cleaning, a self assembled monolayer (SAM) was formed onto the gold-surface of the nanogratings, by derivatization with 300 µM (R)-(+)-α-lipoic acid in ethanol 8% v/v, so to provide carboxylic acids to the surface. After extensive washings in water, the nanogratings were treated for 20 min with 50 mM EDC/NHS (with 1:1 mol:mol) in 10 mM MES pH 5.5, then added of in 10 mM Lys-Lys in 100 mM phosphate buffer (PB) pH 7.4. The nanoMIPs were resuspended at 0.5 mg/ml in 12 mM EDC and 50 mM in 10 mM MES pH 5.5. Aliquots of nanoMIPs were diluted 1:1 in the PB pH 7.4 and placed onto the nanogratings to react for 2 h at room temperature in a sealed humid box. At the completion, the nanoplasmonic platforms were then washed extensively prior to use. The functionalization reaction, the coverage of the nanosurfaces with nanoMIPs was controlled by means of scanning electron microscopy (SEM) and by atomic force microscopy (AFM). Surface topography by AFM was mapped using an NT-MDT Solver Pro system equipped with S7 scanner. All samples were imaged in semi-contact mode using super sharp diamond-like carbon tip (NSG10_DLC, NT-MDT, 1 nm nominal tip radius, typical resonance frequency of 255 kHz), collecting 4 × 4 µm and 1 × 1 µm, 512 points resolution images, acquired on different regions of each sample. AFM data were analyzed with the support of Gwyddion analysis software (Nečas et al., 2012) The AFM and SEM images of the nanoMIP functionalized nanogratigs are reported in Figure 4.

### 2.6 Binding Experiments Onto the NanoMIP-Nanoplasmonic Sensing Chip

Prior to use, the nanoMIPs on the sensing platform were treated with 8% v/v ethanol in water, so to induce their shrinking (Bertolla et al., 2017) and followed by a full rehydration process in MilliQ water (up to 2 h) and by the conditioning of the nanoMIP-nanoplasmonic sensor in 10 mM PBS pH 7.4. Swelling and shrinking of the nanoMIPs on the plasmonic surfaces were monitored as optical shifts. The conditioning is performed just once at the end of the functionalization. Then, a volume of sample of 5 μL was used for the measurements. The measurements were performed in PBS 10 mM pH 7.4 at room temperature. Serial dilutions of BSA in the range from 1 fM to 80 pM were prepared in triplicate and measured. The wavelength minima, or the shift in the wavelength, were plotted as a function of BSA concentration. Isotherms were fitted with Origin 9.0 using Hill equation: y = START + (END − START) * xn/(kn + xn), where START and END were the initial and final y values; x was the concentration of BSA; n was the Hill parameter; k was the half saturation or apparent dissociation constant (EC_50_ or K_Dapp_); the fitting converged satisfying the tolerance criteria. Experiments with competitor proteins (alpha-lactalbumin and myoglobin) were carried out at a fixed concentration of 690 pM. All samples were incubated for the fixed time of 5 min on the sensing platforms prior to measure the wavelength minimum of the optical signal. After each measurements the chip was extensively washed with PBS.

After the regeneration steps, the measurements were repeated. The stdv for the repeated measurements was within the 10%. Moreover, the experimental repeatibility was tested on n = 3 sensor chips, stdv resulted within 10%.

## 3 Numerical and Experimental Results

### 3.1 Numerical Results

**FIGURE 5:**
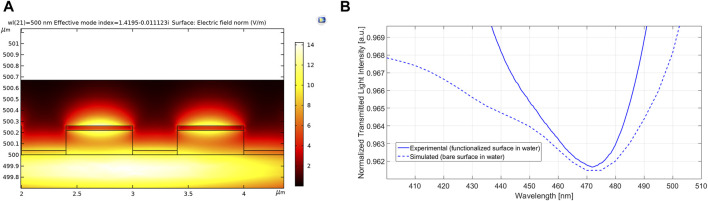
Numerical results based on a mode analysis of the nanoplasmonic sensor chip, obtained by using Comsol Multiphysics. In particular, **(A)** shows the simulated electric field norm; **(B)** reports the normalized transmitted spectrum relative to the fundamental mode, obtained by the normalization of the transmitted spectrum in water with the reference spectrum. In addition, to compare the numerical and the experimental resonance, the spectrum experimentally obtained exploiting the functionalized sensor chip in water has also been reported.

A numerical analysis of the nanoplasmonic probe has been performed by using Comsol Multiphysics. In particular, we have carried out a mode analysis of the sensor chip, similar to (Kadhim et al., 2020). The electric field norm and the normalized transmitted spectrum relative to the fundamental mode are plotted in Figure 5 when the nanograting bare surface (sensors chip without nanoMIPs) is in contact with water (refractive index value equal to 1.332). More specifically, the spectrum plotted in Figure 5B has been carried out by normalization the transmitted spectrum in water with the reference spectrum (relative to the air as a surrounding medium). The simulated spectrum has also been compared with the spectrum experimentally acquired by the functionalized sensor chip in water (blank solution). The resonance wavelength range between the numerical and experimental results is very similar, as shown in Figure 5B.

### 3.2 Experimental Results

The sensing response of the non-optimized nanoplasmonic chip functionalized with the highly responsive nanoMIPs was tested by challenging the platform with increasing concentrations of the target analyte, namely the BSA. In the experimental setup we considered the PMMA substrate of the nanoplasmonic chip as a transparent substrate, onto which the periodic nano-stripes were allocated in a longitudinal orientation with respect to the direction of the input light.

**FIGURE 6:**
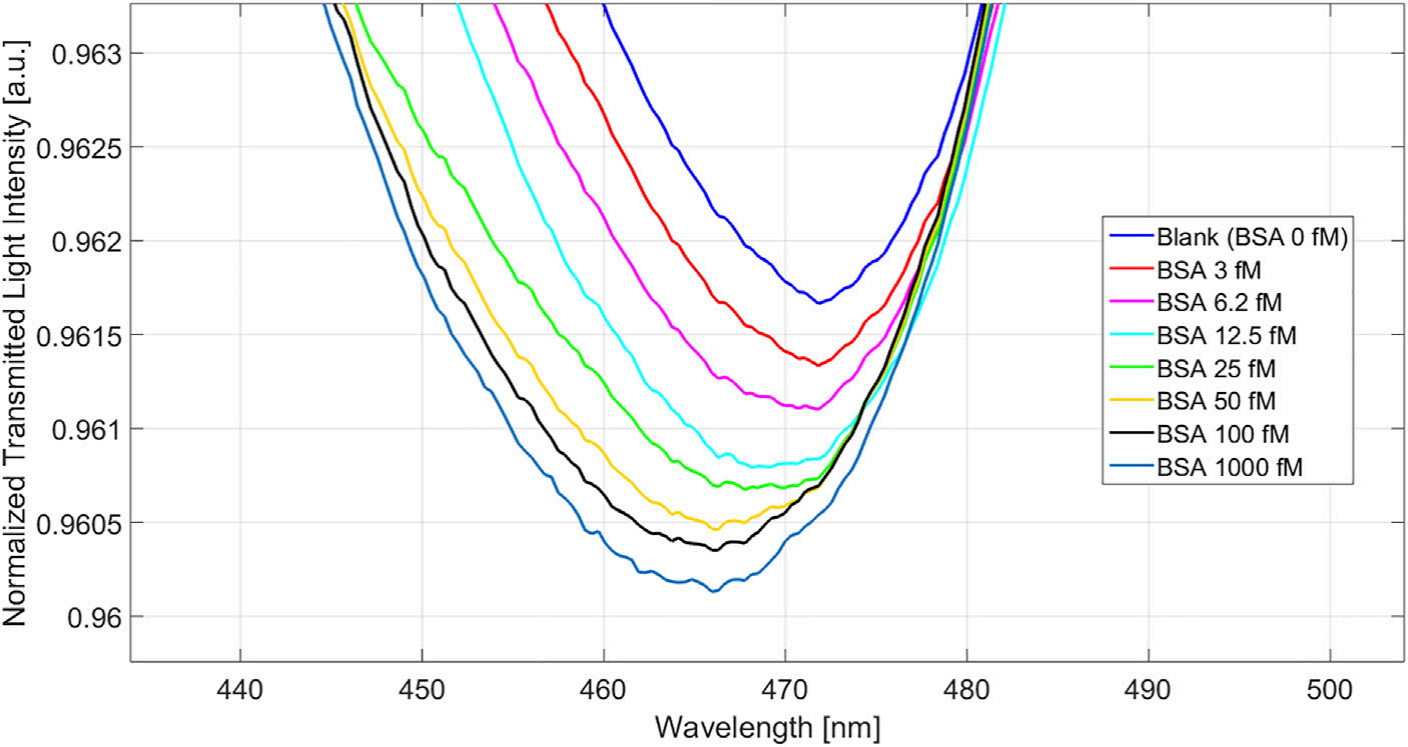
Nanoplasmonic spectra obtained at different concentrations of BSA in buffer solution.

Normalized transmitted spectra obtained at different BSA concentrations, in the wavelength range from about 360 to 910 nm, are shown in SI Supplementary Figure S2, whereas Figure 6 shows the zoom of it in the resonance wavelength region. In particular, Figure 6 shows the normalized nanoplasmonic spectral response of the bio/chemical sensor for BSA concentrations ranging from 3 fM to 1 pM. A clear blue shift of the resonance wavelength (λ) was observed when the analyte concentration increased, in accordance to earlier observations on the blue-shifting of soft, responsive, nanoMIPs as well as of layers of MIP hydrogels upon binding of their target analyte (Matsui et al., 2004; Cennamo et al., 2020).

**FIGURE 7:**
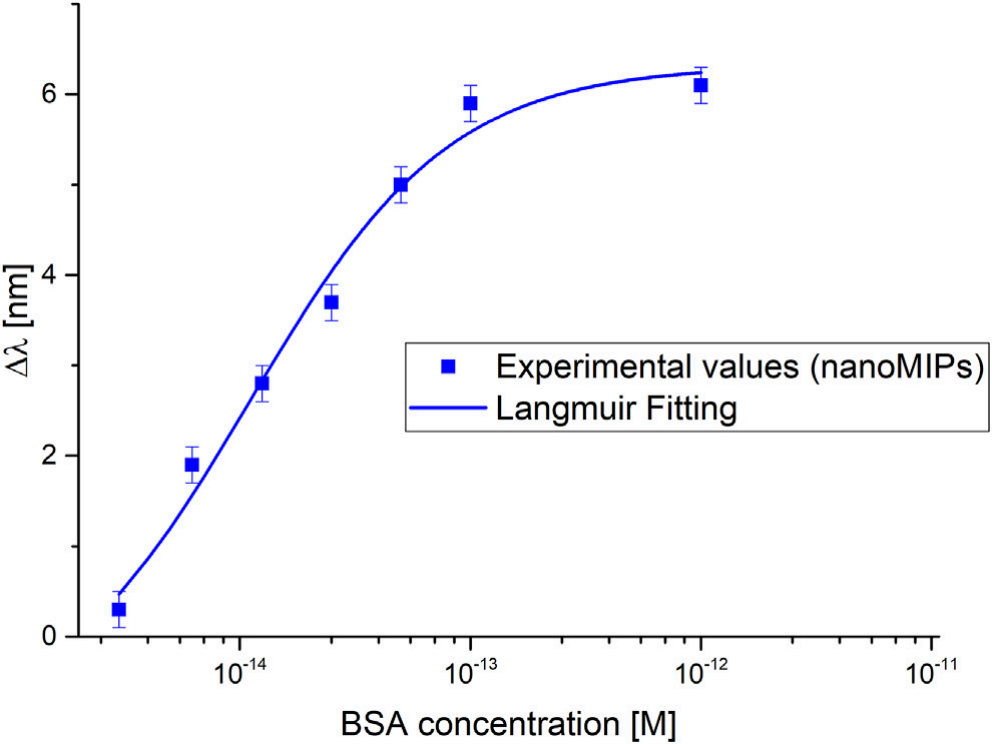
Dose-response curves of the BSA detection in buffer solution exploiting nanoMIPs combined with a not-optimized nanoplasmonic chip.

Figure 7 shows the absolute value of the resonance wavelength shift with respect to the blank (solution without the analyte), along with the Langmuir fitting of the experimental data and the error bars, in a semi-log scale for the tested sensor.

The dose-response curve has been fitted by the Langmuir equation here recalled:

$$\left|\Delta \lambda \right|=\left|\lambda_{c}-\lambda_{0}\right|=\left|\Delta \lambda_{\max}\right|\cdot \left(\frac{c}{\left(K+c\right)}\right)$$ (1)

where c is the analyte concentration, $\lambda_{c}$ is the resonance wavelength at the concentration c, $\;\lambda_{0}$ is the resonance wavelength value at zero concentration (blank), $\Delta \lambda_{max}$ is the maximum value of Δλ (calculated by the saturation value minus the blank value).

As shown in Eq. 1, at low analyte concentration (c), i.e., *c* much lower than K, the Langmuir equation can be considered a linear equation, with sensitivity (slope) |Δλ_max |/K, defined as the “sensitivity at low concentration”. The limit of detection can be approximated as the ratio of two times the standard deviation of the blank and the sensitivity at low concentration (|Δλ_max |/K).

The parameters relative to Langmuir fitting have been reported in Table 1. These values have been achieved by OriginPro software (Origin Lab. Corp., Northampton, MA, United States), and can be used to calculate the chemical parameters of the considered sensor, as reported in Table 2.

**TABLE 1:** Langmuir parameters of BSA detection in buffer solution exploiting the proposed biochemical sensor.

Sensor	∆λ_0_ [nm]		∆λ_max_ [nm]		K [M]		Statistics	
	Value	Standard error	Value	Standard error	Value	Standard error	Reduced Chi-Sqr	Adj. R-square
NanoMIPs combined with a not-optimized nanoplasmonic chip	−1.12	0.68	6.32	0.26	1.10 × 10−^14^	3.1 × 10−^15^	2.417	0.979

**TABLE 2:** Sensor’s chemical parameters for BSA detection in buffer solution.

Sensor	Parameters	Value
NanoMIPs combined with a not-optimized nanoplasmonic chip	*K*_aff_ [ M^−1^ ] (*K*_aff_ = 1/K)	9.07 × 10^13^
	Sensitivity at low *c* [nm/M] (Sensitivity at low *c* = ∆λ_max_/K)	6.75 × 10^14^
	LOD [M] (2*standard deviation of blank/Sensitivity at low *c*)	2.03 × 10^−15^
	Detection range [M]	2 × 10^−15^ – 10 × 10^−14^

As shown in Table 2, the proposed nanoMIPs-based sensor presents superior performances with respect to the sensor configuration based on an optimized nanoplasmonic sensor chip combined with an ultra-thin MIP layer (Arcadio et al., 2021c). So, despite using a non-optimized nanoplasmonic chip, we observed a great improvement of the biochemical sensor’s performance, possibly due to the superior responsivity of the nanoMIP receptors, that are indeed made of soft responsive material (Bertolla et al., 2017). In fact, in the present work, the BSA detection range attained a dynamic range of response of 2 × 10^–15^ – 10 × 10^−14^ M, which was significantly more sensitive than the results earlier achieved onto an optimized nanoplasmonic sensor modified with an ultrathin MIP layer (2.3 × 10^−11^ – 10 × 10^−9^ M), as reported in (Arcadio et al., 2021c).

The enhanced responsivity of the nanoMIP receptors, which are indeed made of “soft” responsive material (Bertolla et al., 2017), is due to the shape-changing of the nanoMIPs in the presence of the binding with the target substances, and so at the significant refractive index variation of the nanoMIPs with the binding.

**FIGURE 8:**
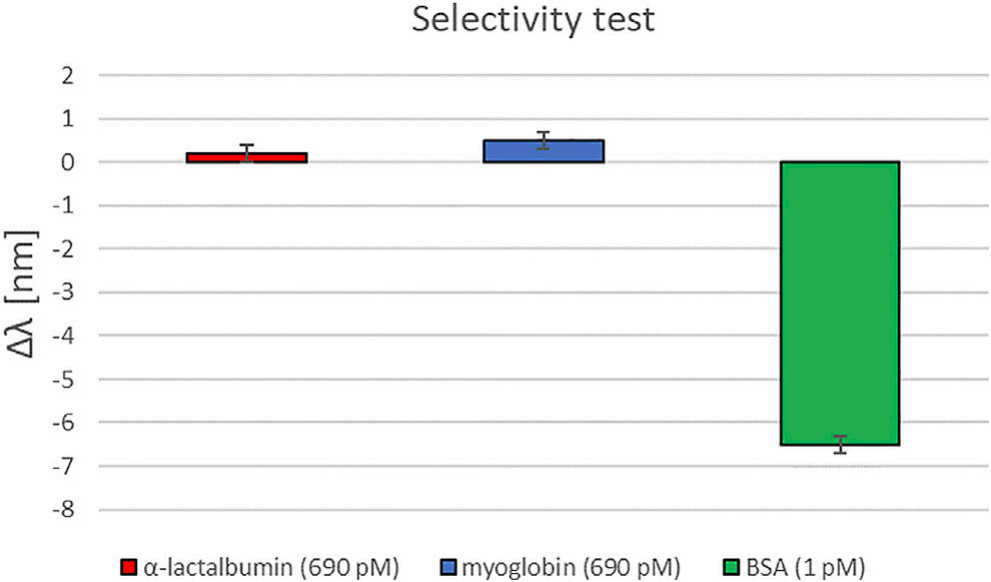
A selectivity test: Resonance wavelength variations obtained by different substances.

Finally, the selectivity test obtained by two different substances, alpha-lactalbumin and myoglobin, are reported in Figure 8. With a concentration of 690 pM, both these substances produced just a small red-shift variation (about 0.5 nm). In contrast, the target analyte (BSA), even at a concentration of 1 pM, produced a blue-shift variation of about 6.5 nm, similarly to the test reported in Figure 7.

## 4 Discussion

**FIGURE 9:**
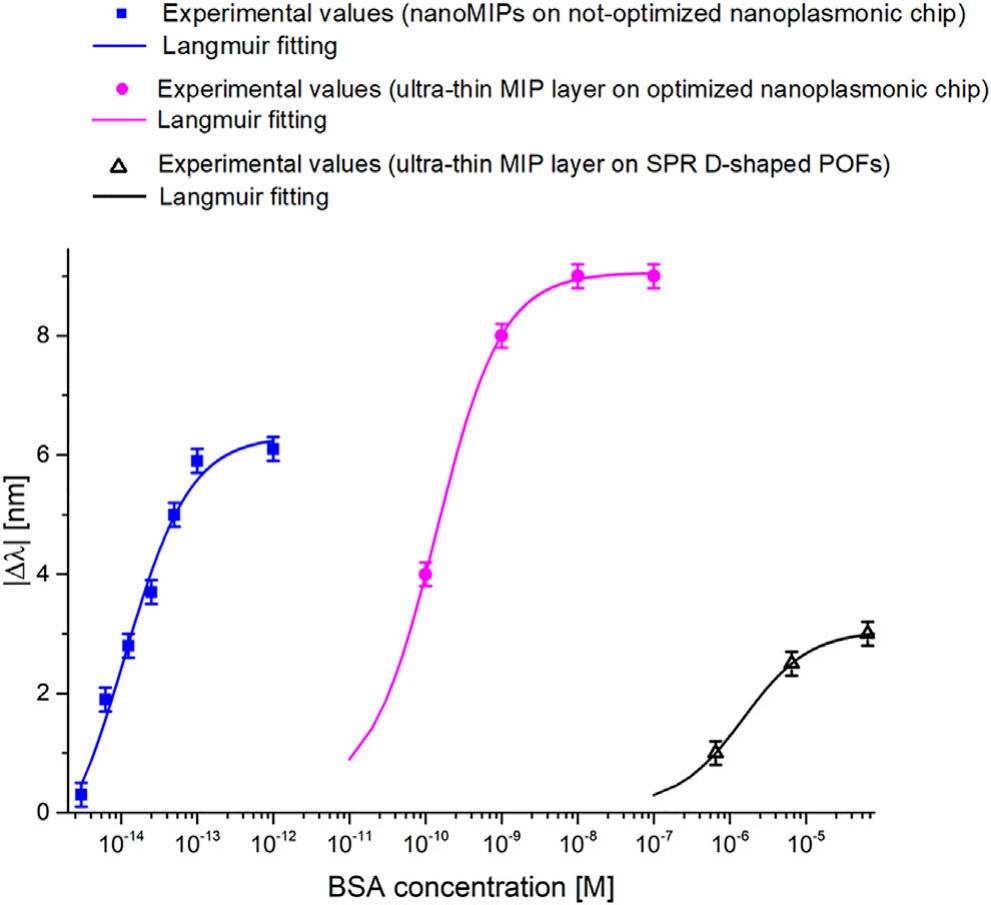
BSA dose-response curves obtained by different sensing approaches based on the combination of plasmonic probes and biomimetic receptors.

This work sheds light on the effect of playing on the combinations of different kinds of receptors with different kinds of plasmonic transducers. The results show that the performance of the sensor device can be tuned according to the specific application, simply by optimizing the combination of sensor’s design, and receptor element. As an example, we already demonstrated in (Arcadio et al., 2021b) that a MIP ultra-thin layer for BSA combined to an optimized nanoplasmonic chip presents a BSA detection range from 37 pM to 100 nM, whereas when the same MIP ultra-thin layer was coupled to an SPR D-shaped POF probe, it produces a markedly different detection range, i.e., from 0.37 to 6.5 µM (Arcadio et al., 2021b; Arcadio et al., 2021c). Here we showed that exploiting soft BSA-specific nanoMIPs with a non-optimized nanoplasmonic chip resulted in the detection window shifted to 2–100 fM. Figure 9 reports an overview of the different BSA detection ranges obtained by combining different types of transducers and MIP receptors. A further consideration relates to the effect of choosing soft nanoMIPs as a receptor. The previously reported data on nanoMIP-functionalized SPR D-shaped POF sensors for the selective detection of human transferrin (Cennamo et al., 2020) and the actual data from the nanoMIPs for BSA detection coupled onto the non-optimized nanoplasmonic chip enabled to achieve femtomolar detection for their analytes, so suggesting that when soft nanoMIPs are the receptor element, the kind of plasmonic probe used to monitor the phenomenon plays a less relevant role. Additionally, to better outline the comparison between the proposed BSA-sensor configuration and the BSA optical sensors found in the literature, Table 3 summarizes the main characteristics of the BSA-sensors.

**TABLE 3:** Characteristics of several sensors’ configurations for the selective detection of BSA.

Configuration	LOD	BSA detection range	Reference
Fluorescence sensor	10 nM	0.01–2 [μM]	Cui et al. (2020)
Aggregation-induced emission biosensor coupled with graphene-oxide	0.4 μM	0.4–1.5 [μM]	Xu et al. (2011)
SPR-MoS_2_ optical fiber	4.36 nM	4.36–750 [nM]	Kaushik et al. (2019)
LSPR based on bimetallic nanoparticles	0.15 pM	0.15–15 ⋅ 10^3^ [pM]	Jia et al. (2014)
SPR D-shaped POFs	0.37 µM	0.37–6.5 µM	Cennamo et al. (2021b)
Optimized GNG-based Monitored exploiting the transparent substrate (different setup)	37 pM	37 pM–100 nM	Arcadio et al. (2021b)
Optimized GNG-based longitudinal (blue-shift resonance)	23 pM	23 pM–10 nM	Arcadio et al. (2021c)
Optimized GNG-based longitudinal (red-shift resonance)	0.54 µM	0.54–10 µM	Arcadio et al. (2021c)
Optimized GNG-based orthogonal	42 pM	42 pM–10 nM	Arcadio et al. (2021c)
Not-optimized GNG-based longitudinal combined with nanoMIPs	2.03 fM	2 fM – 0.1 pM	This work

**FIGURE 10:**
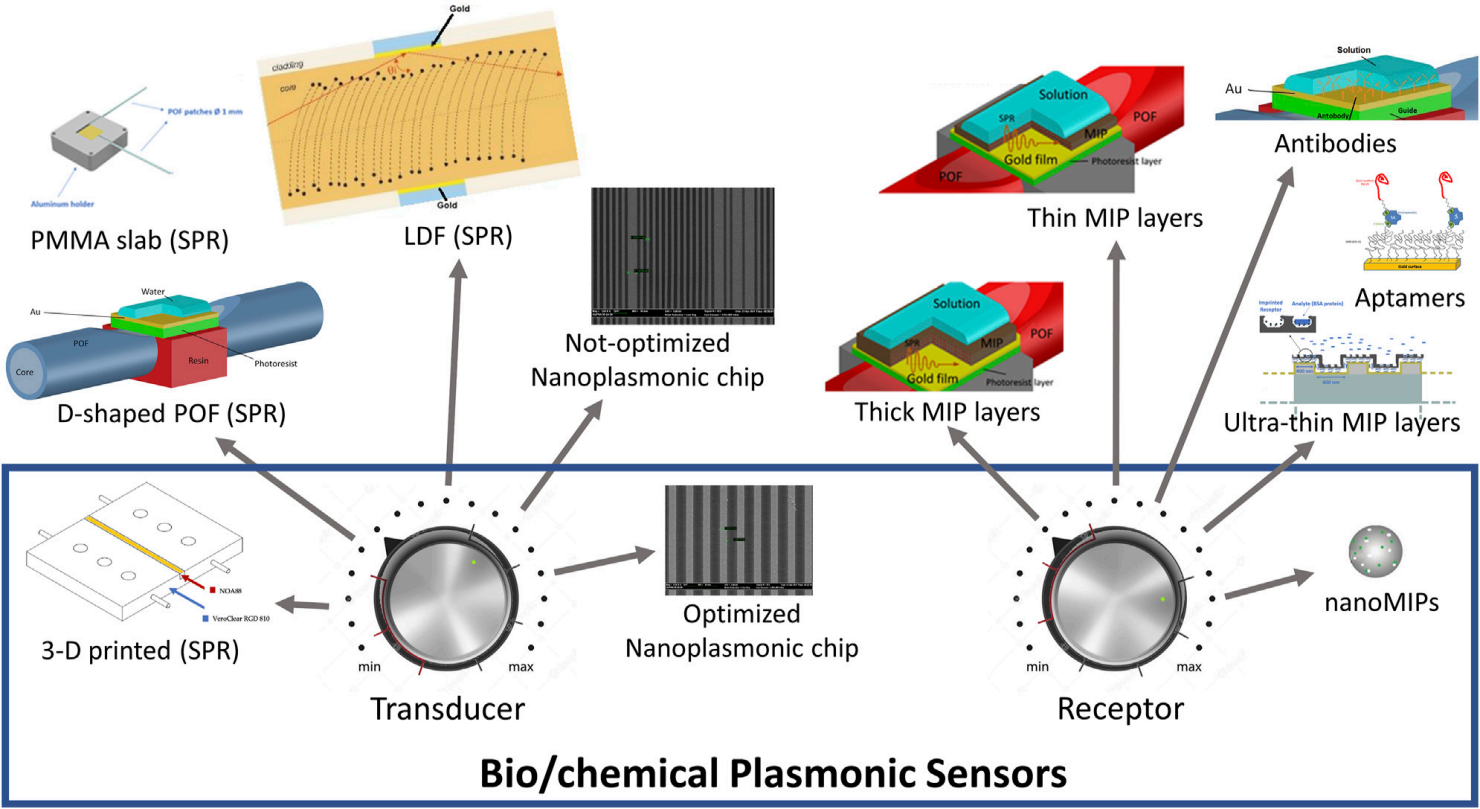
An example of a summary on the designer’s choices of transducers and receptors combinations so to realize custom bio/chemical sensor devices.

In conclusion, our results indicate how the combination of PMMA plasmonic sensing platforms to different kind of receptors, allow to respond to any design specifications, thus offering a general, and widely applicable sensing solution. Figure 10 proposes an overview of how a designer could use the bio/chemical sensor components, both the transducer and the receptor, to modify the response of the sensor system in terms of detection range. In particular, in Figure 10, we report several kinds of plasmonic probes, developed by Cennamo et al. (Cennamo et al., 2017; Cennamo et al., 2011; Cennamo et al., 2018b; Cennamo et al., 2021a; Arcadio et al., 20121b; Arcadio et al., 2021c), and different types of receptors, combined with plasmonic probes (Cennamo et al., 2019b; Cennamo et al., 2020; Zeni et al., 2020; Arcadio et al., 2021b; Arcadio et al., 2021c; Pesavento et al., 2021). The balance of the combination of these two components would produce the desired optimal performance. For example, the three BSA dose-response reported in Figure 9 were obtained by the following configurations reported in Figure 10: two by coupling the receptor named “Ultra-thin MIP layer” with two different plasmonic probes (transducer), named “D-shaped POF (SPR)”, and “Optimized Nanoplasmonic chip”, and one by coupling the receptor “nanoMIPs” with the plasmonic probe named “Non-optimized Nanoplasmonic chip”.

## 5 Conclusion

We developed and tested a novel bio/chemical sensor based on nanoMIPs combined to a nanoplasmonic sensor chip. The sensor configuration herein proposed enabled to decrease the LOD for the tested analyte (BSA) of four orders of magniture, i.e., from 23 pM to 2 fM. The inherent soft and deformable character of the nanoMIP receptors is hypothesized to be responsible to attain such low detection limits, despite, in the tested sensor, the nanoMIPs were layered onto a non-optimized nanoplasmonic chip, this latter being characterized by about a half value of the bulk sensitivity with respect to the optimized pattern (Arcadio et al., 2021b; Arcadio et al., 2021c). Moreover, the proper combinations of plasmonic probes and receptors permit to achieve detection ranges specific for any application.

## Data Availability

The raw data supporting the conclusion of this article will be made available by the authors, without undue reservation.
