# Supplementary material for: On the Effect of Soft Molecularly Imprinted Nanoparticles Receptors Combined to Nanoplasmonic Probes for Biomedical Applications
Source: Front Bioeng Biotechnol. 2021 Dec 21;9:801489. doi: 10.3389/fbioe.2021.801489 (PMC8724520; doi:10.3389/fbioe.2021.801489)

On the Effect of Soft Molecularly Imprinted Nanoparticles Receptors combined to Nanoplasmonic Probes for Biomedical Applications

Nunzio Cennamo[1,§], Alessandra Maria Bossi[2,§], Francesco Arcadio[1], Devid Maniglio[3], Luigi Zeni[1,*]

[1] Department of Engineering, University of Campania Luigi Vanvitelli, Via Roma 29, 81031 Aversa, Italy
[2] Department of Biotechnology, University of Verona, Strada Le Grazie 15, 37134 Verona, Italy
[3] University of Trento, Department of Industrial Engineering, Via Delle Regole 101, 38123 Trento, Italy

[§]These authors have contributed equally to this work and share first authorship
*Corresponding author: luigi.zeni@unicampania.it

Supplementary Information

1. **Size of the nanoMIP by dynamic light scattering.**

SI Figure 1. Dynamic light scattering of the nanoMIPs.

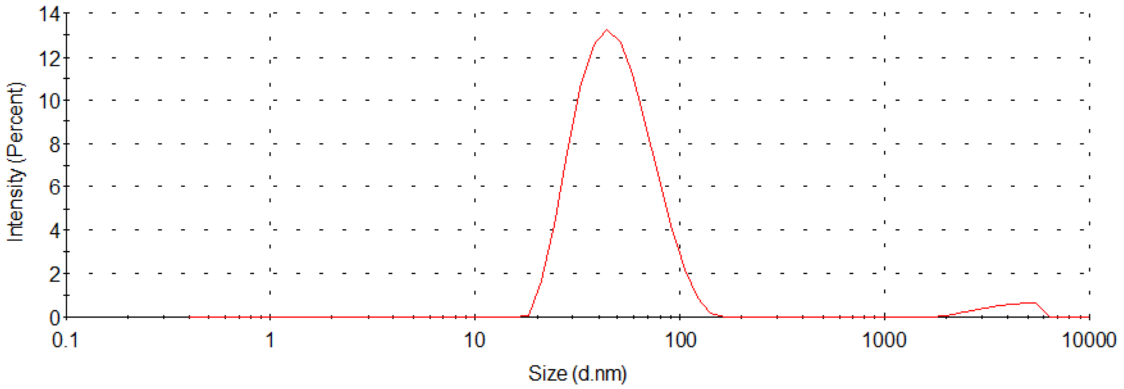

2. Full spectra of the nanoMIP-nanograting sensor's response to BSA.

SI Figure 2. Normalized transmitted spectra at different BSA concentrations in the wavelength range from about 360 nm to 910 nm.

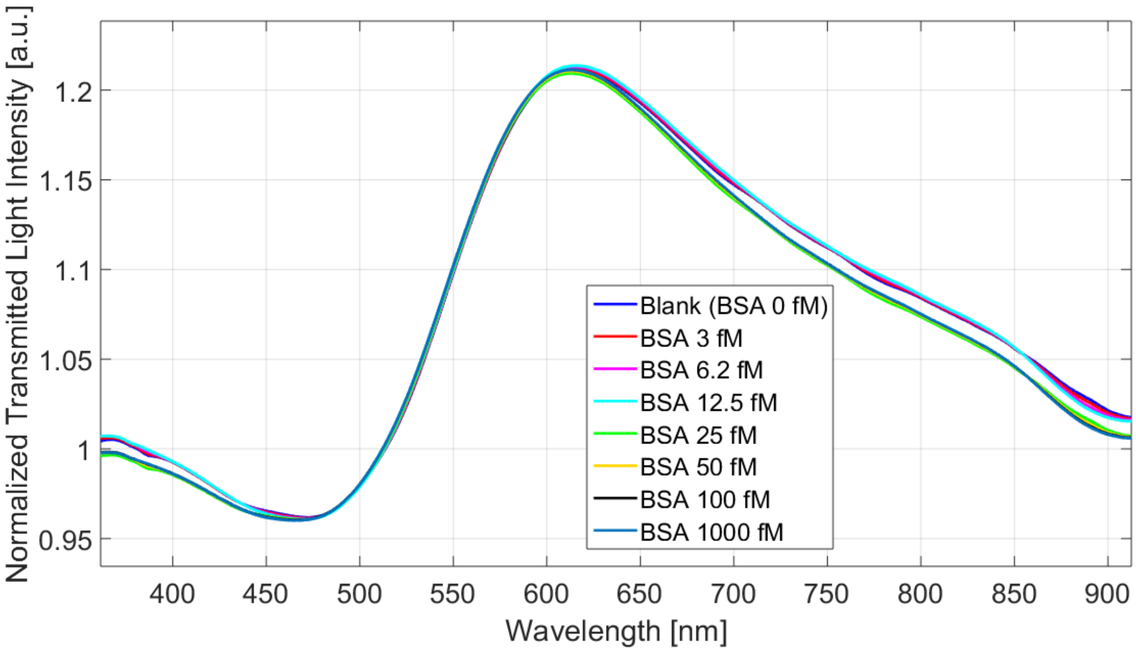

Supplement: Supplementary file 1 [file DataSheet1.pdf]
